# A Novel Optical Fiber Terahertz Biosensor Based on Anti-Resonance for the Rapid and Nondestructive Detection of Tumor Cells

**DOI:** 10.3390/bios13100947

**Published:** 2023-10-23

**Authors:** Zhe He, Yueping Luo, Guorong Huang, Marc Lamy de la Chapelle, Huiyan Tian, Fengxin Xie, Weidong Jin, Jia Shi, Xiang Yang, Weiling Fu

**Affiliations:** 1 Department of Laboratory Medicine, Southwest Hospital, Army Medical University (Third Military Medical University), Chongqing 400038, China; hezhe957467915@163.com (Z.H.); yollowrong@sina.com (G.H.); marc.lamydelachapelle@univ-lemans.fr (M.L.d.l.C.); thylcq@163.com (H.T.); xiefengxin0310@163.com (F.X.); jinwd14@lzu.edu.cn (W.J.); 2 Tianjin Key Laboratory of Optoelectronic Detection Technology and System, School of Electronic and Information Engineering, Tiangong University, Tianjin 300387, China; 2131070920@tiangong.edu.cn; 3 Institut des Molécules et Matériaux du Mans (IMMM-UMR CNRS 6283), Université du Mans, Avenue Olivier Messiaen, 72085 Le Mans, France

**Keywords:** terahertz, optical fiber, tumor cells, anti-resonant effect

## Abstract

The sensitive and accurate detection of tumor cells is essential for successful cancer therapy and improving cancer survival rates. However, current tumor cell detection technologies have some limitations for clinical applications due to their complexity, low specificity, and high cost. Herein, we describe the design of a terahertz anti-resonance hollow core fiber (THz AR-HCF) biosensor that can be used for tumor cell detection. Through simulation and experimental comparisons, the low-loss property of the THz AR-HCF was verified, and the most suitable fiber out of multiple THz AR-HCFs was selected for biosensing applications. By measuring different cell numbers and different types of tumor cells, a good linear relationship between THz transmittance and the numbers of cells between 10 and 10^6^ was found. Meanwhile, different types of tumor cells can be distinguished by comparing THz transmission spectra, indicating that the biosensor has high sensitivity and specificity for tumor cell detection. The biosensor only required a small amount of sample (as low as 100 μL), and it enables label-free and nondestructive quantitative detection. Our flow cytometry results showed that the cell viability was as high as 98.5 ± 0.26% after the whole assay process, and there was no statistically significant difference compared with the negative control. This study demonstrates that the proposed THz AR-HCF biosensor has great potential for the highly sensitive, label-free, and nondestructive detection of circulating tumor cells in clinical samples.

## 1. Introduction

The sensitive and accurate detection of tumor cells is essential for improving cancer survival rates and successful cancer therapy. Currently, the traditional laboratory identification methods are not perfect due to their different shortcomings. Among these, flow cytometry enables high-throughput single-cell analysis but requires skilled experimenters [1]. Common immunofluorescence-based methods inevitably lead to the loss of cellular activity and biological information [2]. Although quantitative (real-time) reverse transcription polymerase chain reaction (qRT-PCR) is highly sensitive, it still involves from complex procedures and cannot be used to obtain morphological information [3]. In addition, gene sequencing is costly and requires skilled operators that are not suitable for cancer screening [4]. Therefore, developing a sensitive and nondestructive method for tumor cell detection would be significant due to its potentially vital applications in clinical laboratories.

Terahertz (THz) waves are defined as electromagnetic waves with frequencies ranging from 0.1 to 10 THz (3000–30 μm), which are located between the high-frequency microwave and far-infrared regions [5]. THz waves are widely used in biomedical fields such as biosensing [6], medical imaging [7], and pharmaceuticals [8] due to their fingerprint characteristics, low photon energy, and easy operation. THz differential time-domain spectroscopy (THz-DTDS) was used to measure small changes in bovine pulmonary microvascular endothelial cells after the addition of vascular endothelial growth factor (VEGF), which were not observed under light microscopy [9]. Later, it was found that THz time-domain spectroscopy (THz-TDS) can be used to monitor cell activity and study the hydration status inside tumor cells in real time [10]. Caroline B et al. measured the THz spectral characteristics of components in human whole blood and observed a linear relationship between red blood cell concentration and THz signal [11]. Cao et al. used THz time-domain attenuated total reflection spectroscopy (THz TD-ATR) to measure aqueous solutions containing colorectal cancer cell lines and observed that cancer cells have different spectral characteristics compared to normal cells and that there is no linear relationship between cell concentration and absorption coefficient. Subsequently, they used principal component analysis (PCA) and random forest (RF) methods to identify different tumor cell lines, demonstrating the potential of THz waves to be applied in liquid biopsies [12,13]. Although the above methods provide measurements of cell viability, cell state, and concentration, there are some shortcomings in the current clinical application of these techniques, such as their low sensitivity, lack of characteristic peaks, lack of suitability for suspension cells, and large sample demand. THz waves are highly sensitive to water and coupled to atmospheric components, so the free space transmission of THz waves experiences significant energy loss [14]. As a result, THz-based biosensors are hindered in real liquid sample detection.

Hollow-core fibers (HCFs) have attracted attention among researchers in recent years. Because of their advantages of extremely low absorption loss, simple structure, bending losses, and low dispersion [15], HCFs are widely used in communication [16], sensing [17], and imaging [18]. In sensing applications, HCFs are used to increase the THz power interaction with the analyte, resulting in an increase in the sensitivity of the sensor, which can then been applied for gas [19], biological [20], and chemical sensing [21]. Md et al. designed a hollow core Photonic Crystal Fiber (HCPCF) to detect blood components. Under optimal conditions, the simulation results showed a relative sensitivity to the components in the blood of more than 90% [22]. Mohammad et al. proposed a THz hollow core Topas-based photonic crystal fiber (PCF) biosensor and achieved a sensitivity for RBC of 99.39%, a sensitivity for hemoglobin of 99.27%, a sensitivity for WBC of 99.12%, and a sensitivity of plasma is 99.03% at optimum design conditions [23]. A novel hollow core photonic crystal fiber (PCF)-based hollow core optical waveguide was used to distinguish various tumor cells, and it achieved an extremely high relative sensitivity of almost 98% at 2.5 THz with negligible loss [24]. A square core PCF biosensor for the detection of tumor cells as analytes was proposed by Sapana et al. The relative sensitivity of the studied PCF model sensor was 81.38% for tumor cells and 65.83% for normal cells [25]. Zhang et al. designed a microstructure fiber (MSF) biosensor with a porous core structure that showed a relative sensitivity to breast cancer cells of up to 99.8% under the optimal conditions of 0.9 THz [26]. Although the THz fiber biosensors proposed above have extremely high relative sensitivity, they have not been tested in biological samples, and the relationship between sample concentrations and THz signals needs to be clarified before their clinical application.

Herein, we describe the design of a THz anti-resonance HCF (AR-HCF) biosensor for the detection of tumor cells. An anti-resonant fiber has lower transmission loss by inhibiting the coupling of core and cladding modes. In addition, AR-HCF has the characteristics of a high reflectivity, wider transmission band, and low dispersion [27], which are particularly suitable for the field of biosensing and in vitro diagnosis. The AR-HCF is made of photosensitive resin and was created via 3D printing. We manufactured THz AR-HCFs with different cladding thicknesses and fiber diameters and selected the suitable structural parameters through the simulation results. By detecting different concentrations and different kinds of biosamples, it was proven that this optical fiber has good sensitivity and specificity in tumor cell detection. Due to its nondestructive nature and rapid assay within 10 s, the biosensor is compatible with immunofluorescence, sequencing, qRT-PCR, and other laboratory methods, providing more biological information. This THz fiber biosensor has great potential applications in the context of early-stage tumor diagnosis.

## 2. Materials and Methods

### 2.1. Materials and Reagents

Fetal bovine serum (FBS), phosphate-buffered solution (PBS), penicillin-streptomycin solution, 0.25% trypsin containing 0.02% ethylenediaminetetraacetate (EDTA), Leibovitz’s L-15 medium, and RPMI 1640 medium were purchased from Gibco, Life Technology, Shanghai, China. Propidium iodide (PI) was obtained from Invitrogen, Carlsbad, CA, USA). All of the disposable consumables, such as cell culture flasks, mesh filters (45 μm), suction tips, pipettes and tubes, used in the experiment were provided by Nest Biotechnology, Wuxi, China.

### 2.2. Fabrication of THz AR-HCFs

We used photopolymer 3D printing technology to achieve economic and efficient manufacturing, using on a commercial 3D printer (ANYCUBIC) and commercially available photosensitive resin. When printing, the stereolithography appearance (SLA) printer uses an inverted lithography setting for the layer-by-layer curing of the fiber. A 405 nm matrix light source was used to provide selective voxel exposure for each layer. After printing, the fiber was washed with water and 95% alcohol to remove excess uncured resin. Layer-by-layer selective voxel crosslinking was adopted to ensure structure accuracy during cleaning and post curing. In addition, the physical simulation requires the use of the characteristic parameters of the material. The structural parameters of twelve are as follows: core diameter set as 2 mm, 3 mm, and 4 mm; semimajor axes of the semielliptical tubes set as 3 mm, 4 mm, and 5 mm; semiminor axes of the semielliptical tubes set as 2 mm, 3 mm, and 4 mm; and wall thickness set as 0.6 mm, 0.8 mm, and 1 mm, respectively. The Vernier caliper results proved that the morphology of the 3D-printed waveguide was consistent with the design. According to the quantitative analysis of each waveguide, the print error range of the core diameter size was −27~26 µm. The printing error ranges of the wall thickness and semiminor axis length were −20~23 µm and −25~27 µm, respectively. The difference between the measured and designed dimensions is within the allowable printer error range (<50 µm).

### 2.3. Cell Culture

Human breast cancer cell lines (MDA-MB-453, MDA-MB-231, and BT-474) with authenticated STR loci were purchased from Procell Biotech. (Wuhan, China). All cells were cultured in suitable medium containing 10% FBS and 1% penicillin-streptomycin in a humidified cell incubator at 37 °C with 5% CO_2_. MDA-MB-453 and MDA-MB-231 cells were cultured using L-15 medium, and BT-474 cells were cultured using Roswell Park Memorial Institute (RPMI)-1640 media.

### 2.4. Cell Suspension Preparation and Measurement

The cells were digested with 0.25% trypsin containing 0.02% EDTA when they reached approximately 80% confluence. Then, the cells were washed with PBS and resuspended in PBS. Ten microliters of cell suspension was placed on a cell counter plate and counted three times under a cell counter, and the mean value was taken as the number of cells. Based on the counting results, the number of cells in the 100 µL cell suspension was adjusted to 1 × 10^6^. Then, cell suspensions with varying cell numbers were obtained via carrying out multiple dilutions. The final numbers of cells in the 100 µL cell suspension were 1 × 10^6^, 1 × 10^5^, 1 × 10^4^, 1 × 10^3^, 1 × 10^2^, and 1 × 10. A 100 μL sample was added equally to four envelopes, each containing 25 μL of sample. Then, the HCF was placed into the detection chamber to obtain the THz transmission spectra. The THz transmission spectra of the THz AR-HCF and samples were obtained using a commercial THz-TDS system (TAS7500SP, Advantest Co., Tokyo, Japan) with a spectral resolution of 7.6 GHz at room temperature and dry air. The effective frequency region ranged from 0.1 to 4.0 THz. First, the optical fiber was fixed to the test stand, and the transmission spectra of the optical fiber without the sample were recorded. Then, the samples were added successively as mentioned above to obtain the transmission spectra of the samples with different cell numbers. Each sample was measured three times. After the last sample was tested, the fiber was cleaned with ultrapure water and dried before being added to the next sample.

### 2.5. Morphology of the Cells

When the MDA-MB-453, MDA-MB-231, and BT-474 cells were adherent to the wall, we removed them from the medium and then washed them again with PBS to remove the dead cells before finally placing them under an inverted microscope to observe the morphology of the cells.

### 2.6. Simulation

A series of simulations of the AR-HCF biosensor and surface electric field distribution were carried out using COMSOL Multiphysics software 5.6 based on finite element analysis (FEA).

### 2.7. Flow Cytometry

After taking measurements, all cell suspensions of the BT-474, MDAMB-453, and MDA-MB-231 cells were recovered with pipettes and then incubated with PI at room temperature in the dark for 10 min to test cell activity. The BT-474, MDA-MB-453, and MDA-MB-231 cells that were not tested served as negative controls. After incubation, all the samples were washed twice with PBS and then suspended in PBS. Finally, all the samples were analyzed using a BD LSRFortessa flow cytometer following filtration through mesh filters (45 μm) to filter off cell clumps. The results were analyzed using FlowJo software and Origin 2018.

### 2.8. Statistical Analysis

All quantitative data are represented as the mean ± standard deviation (SD) of three or more independent experiments. When the number of experimental groups was 2, the results were analyzed using a *t*-test. All of these analytical methods were performed using SPSS statistic 26 (SPSS Inc., Chicago, IL, USA). *p* < 0.05 was considered significant.

## 3. Results

### 3.1. Theory of THz AR-HCFs

Based on the guidance mechanism, two types of HCFs are proposed. The first type of HCF is a photonic bandgap (PBG) fiber. The second type of HCF is an AR-HCF.

The optical guiding mechanism of an HCF can be explained according to the antiresonant reflecting optical waveguide (ARROW) model. It is also known that there are multiple beam interferences in the cladding of the HCF. The thickness of the high RI cladding determines its optical properties, which is considered a Fabry-Perotetalon. Light leaks out of the HCF when the frequency of the light satisfies the resonant condition of the cladding. Conversely, light that does not satisfy the resonance condition will be trapped and propagate forward in the hollow core. For the proposed THz AR-HCF, the resonant frequencies (*f_m_*) of the fundamental mode can be described as follows [28]:

(1) $$f_{m}=\frac{mc}{2t\sqrt{n_{\mathrm{clad}}^{2}-n_{\mathrm{core}}^{2}}}$$

where *c* is the speed of light, *t* is the thickness of structs surrounding the fiber core, and *n_clad_* represents the refractive index of the cladding material, which, in the context of this work, is UV-resin. The *n_core_* indicates the refractive indices of the core material, and *m* is an integer that represents the order of resonance. Conventionally, the period of the resonant frequency, or the interval between two adjacent resonant frequencies, is treated as the bandwidth of each transmission band.

**Figure 1:**
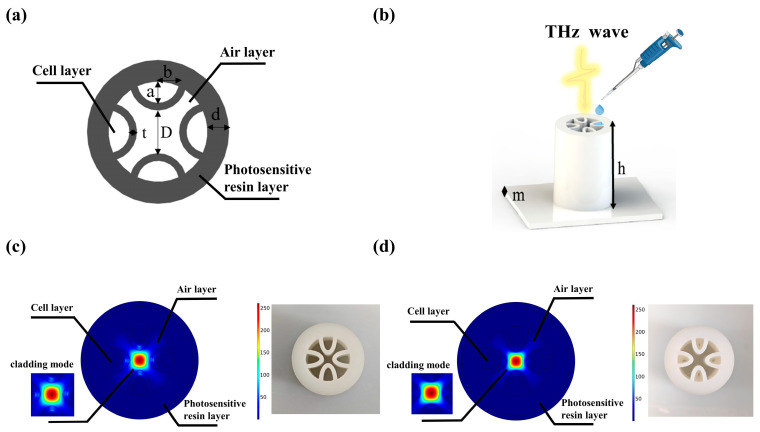
(**a**) Cross-section of a THz AR-HCF. (**b**) Schematic description of a THz AR-HCF for tumor cell detection. (**c**) Electric field distribution and physical object of THz AR-HCF without sample. (**d**) Electric field distribution and physical diagram of the THz AR-HCF after adding PBS.

The cladding design used in the waveguides reported in this paper consists of semielliptical membranes that have been shown to offer low loss at optical frequencies [29]. Figure 1a illustrates the geometry of the cross-section of the structure proposed in this study, along with the definition of the main design parameters that may be modified. The blackish gray region is the photosensitive resin, which is a commonly used material for 3D printing. The core diameter of the proposed fiber is represented by D; the semimajor and semiminor axes of the semielliptical tubes are represented by a and b, respectively; the wall thickness of the semielliptical tubes is represented by t, and the wall thickness of the outer circular tube, which works as a protective layer, is represented by d. Nine samples of 2 cm long fibers were made using a 3D printer using a photosensitive resin. A 3D-printed sample is shown in Figure 1b. A 100 μL sample was added equally to four envelopes, each containing 25 μL of sample. Then, the HCF was placed into the detection chamber to obtain the THz transmission spectra. For the physical simulation, we used COMSOL Multiphysics software 5.6 based on the finite element method for modeling. A perfectly matched layer (PML) boundary was set outside the fiber domain to ensure that the calculated fiber modal characteristics were sufficiently accurate. A nonuniform mesh was used in the model. We chose a smaller mesh size in the anti-resonance tube section compared to the air region. The thickness of the PML was set to 10% of the entire fiber radius, and this does not affect the calculation accuracy. The strong coupling effect between the core and cladding modes can be clearly seen from the mode field profiles of the fiber without the sample in Figure 1c. Due to the strong coupling between the core and cladding modes, the waveguide is highly lossy. Figure 1d shows the fiber with the sample added. However, the frequency is a frequency out of resonance. Since the power is proportional to the squared modulus of the electric field, it is clear that the THz power is well confined in the central air core. This is attributed to the anti-resonant reflection occurring in the core–cladding interface. The physical cross-section and simulation results of the proposed THz optical fiber sample are in Figure 1c,d. The resonant frequencies are calculated for different tube thicknesses by Equation (2). With the tube thicknesses of *t* set as 0.6, 0.8, and 1 mm, the resonant frequencies are obtained as 0.246, 0.327, and 0.409 THz, respectively. In this work, the designed THz fibers are characterized in the frequency range of 0.15–0.80 THz. The transmission loss of the fiber is determined by both confinement loss and effective material loss, which are expressed as follows [30,31,32]:

(2) α=8.686×2πfcIm(neff)+ε0μ0∫matnmatE2αmatdA∫allSZdA

where *f* represents the operating frequency, *c* is the speed of light in vacuum, Im(*neff*) indicates the imaginary part of the complex RI of the fundamental mode, *ε*_0_ and *μ_0_* are the permittivity and permeability under vacuum conditions, respectively. The denominator is integrated over the entire fiber region, including the material region and the hollow tube region, The material parameter of *n_mat_* is the RI and *α_mat_* is the bulk absorption loss of the photosensitive resin. *S_z_* indicates the *z* component of the Poynting vector $S_{z}=\frac{1}{2}(E\times H*)z$ ; here, *E* is the electric field component, and H* is the component conjugate of the magnetic field component. The denominator is integrated over the entire fiber region, including the material region and the hollow tube region.

### 3.2. Design and Analysis of the THz AR-HCF

**Figure 2:**
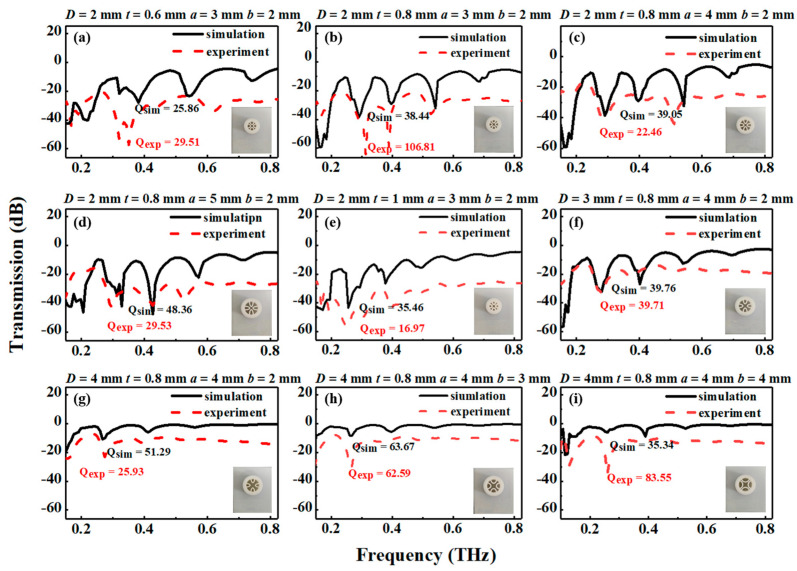
Measured and simulated results for different THz AR-HCFs: (**a**) 2-0.6-3-2; (**b**) 2-0.8-3-2; (**c**) 2-0.8-4-2; (**d**) 2-0.8-5-2; (**e**) 2-1-3-2; (**f**) 3-0.8-4-2; (**g**) 4-0.8-4-2; (**h**) 4-0.8-4-3; (**i**) 4-0.8-4-4. The inset presents the nine THz AR-HCFs. The red dashed line represents the measured results of the AR-HCFs, and the black solid line represents the simulation results of the AR-HCFs.

To analyze the performance of the THz AR-HCF, the transmission loss of the fiber is determined by both confinement loss and effective material loss. Figure 2 shows the simulated demo of the designed AR-HCF with the transmission loss of the fiber for different frequencies following Equation (2). In the design of the fiber, the resonant frequency can be obtained by Equation (1), and it corresponds to the transmission loss of the fiber. The influence of structural parameters on transmission loss will be further analyzed. 

In order to select the most suitable THz AR-HCF biosensor, the THz AR-HCFs with different structural parameters were measured and simulated. In this section, the core diameter, the thickness of the semielliptical layer, and the semiminor axes of the semielliptical tubes are analyzed. The total loss spectra of the representative mode (HE11) of the experiment and the simulation of the nine semielliptical tube fibers are also mentioned.

The numerical and experimental transmission spectra are shown in Figure 2. There are three peaks with transmission loss in the T_0_ order diffraction spectrum from 0.2 THz to 0.6 THz, which shows a good agreement between the experimental and simulated results. A lower loss can be obtained with a larger core diameter. Instead, when the core diameter is increased, the fiber’s single mode characteristic is worse; however, the fiber loss is lower, as shown in Figure 2c,f,g. Similarly, the transmission losses at the second resonance decrease with increasing core diameter. When the wall thickness and semiminor axes of the semielliptical tubes are fixed, the resonant frequency is constant with different core diameters. 

In the process of simulation, the wall thickness is selected to be 0.6 mm, 0.8 mm, and 1.0 mm. As the wall thickness increases, the losses of resonant peak 1 increase, as shown in Figure 2a,b,e. For a tube thickness of 0.8 mm, the maximum loss at the order 2 resonance occurs at approximately 0.28 THz; then, the resonant frequency is shifted to a shorter frequency with increasing tube thickness. As the wall thickness of the elements increases, we note a distinct narrowing of the antiresonance window. Therefore, it can be concluded that the simulation and experimental results agree with the theoretical results.

Figure 2g–i show the influence of the semiminor axes of the semielliptical tubes on the total loss as the semiminor axis length increases from 2 mm to 4 mm for fibers with different ellipticities (a/b). As the curvature of the elements increases, we note a distinct narrowing of the antiresonance window. The curvature of the elliptic tube affects both the resonant frequency and loss peak. This phenomenon is consistent with the assumption that the distance from the geometric center to the wall of the elliptical tube will affect the resonant frequency. Instead, Figure 2b–d show that lower loss can be obtained with smaller semimajor axes of the semielliptical tubes.

To quantitatively evaluate the sensing performance of the fiber, the *Q*-factor can be calculated by using the following equation [33]:

(3) $$Q=\frac{\omega_{r}}{FWHM}$$

where *ω_r_* is the central resonant frequency, and *FWHM* is the full width at half max of the resonance spectrum. The performance of the sensor is both decided by *Q*-factor and peak loss. The greater the *Q*-factor is, the greater the sensitivity of the sensor. According to the experimental results of the AR-HCFs, there are multiple resonance peaks in the range of 0.2 THz. The *Q*-factor of the first resonance peak is the largest, so the change in the first resonance peak during the measurement is mainly observed. Limited by the detection range of the instrument, the result of the fiber with a high *Q*-factor may exceed the detection range of the THz-TDS system. Therefore, the fiber of Figure 2f instead of the fiber with a larger *Q*-factor was ultimately chosen. The differences in the *Q*-factor arise due to the resonant modes with different transmission losses at the resonant frequency.

### 3.3. Sensitivity of the THz AR-HCF Biosensor

**Figure 3:**
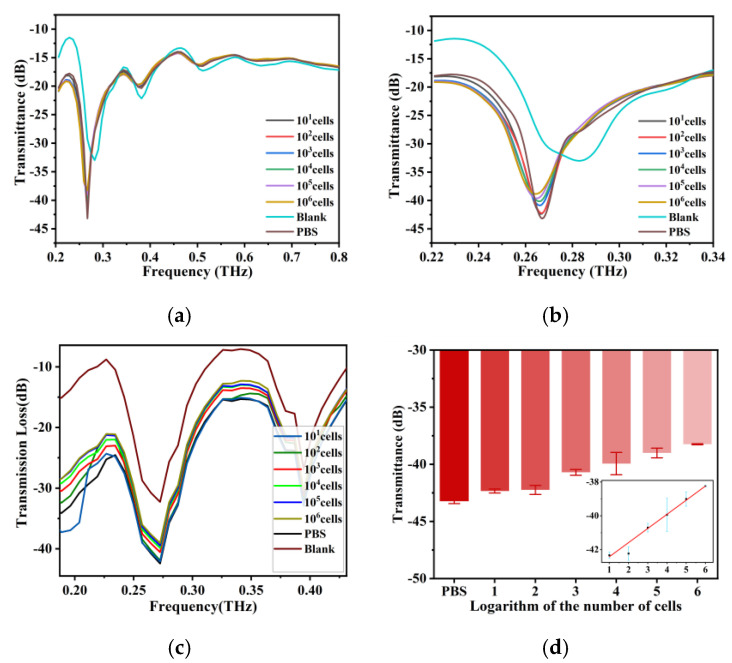
(**a**) THz transmission spectra of BT-474 cell solutions at cell numbers from 10 cells to 1 × 10^6^ cells and the THz AR-HCF without adding the sample between 0.2 THz and 0.8 THz. (**b**) Amplification of the signal from 0.22 THz to 0.34 THz of (**a**). (**c**) Simulation results of THz transmission spectra of fiber with cell numbers from 0 to 10^6^ BT-474 cells and without adding samples. (**d**) THz wave transmittance at 0.26 THz for BT-474 at different cell numbers. The inset shows the linear fit of the logarithm of cell number and THz wave transmittance. Error bars indicate the SD (n = 3).

We measured unsampled optical fibers and samples containing 0–10^6^ BT-474 cells. After PBS was added, the formant of the fiber produced a frequency shift to the left. In Figure 3a, we can see the same THz transmission spectrum as the previous simulation results, along with multiple resonance peaks; so, we chose the resonance peak at approximately 0.26 THz as the characteristic peak. As shown in Figure 3b, after adding the samples, there was a characteristic peak at 0.26 THz, and the transmission gradually increased with increasing cell number, which was consistent with the simulation results (Figure 3c). Figure 3d shows a good linear relationship between the logarithm of cell number and THz wave transmittance in the range of cell numbers from 10 to 10^6^. The linear fitting equation is T (Transmittance) = 0.8365Log(C) − 43.26, and the correlation coefficient is 0.9945. This indicates that the biosensor can quickly detect tumor cells without labels and that it has good sensitivity. The change in transmittance that follows the change in cell number is because as the cell number increases, the number of cells in the sample increases, resulting in a decrease in the water content in the same volume of the sample. Because water is highly absorbent to THz waves, the transmittance increases as the water content decreases.

### 3.4. Specificity of the THz AR-HCF Biosensor

**Figure 4:**
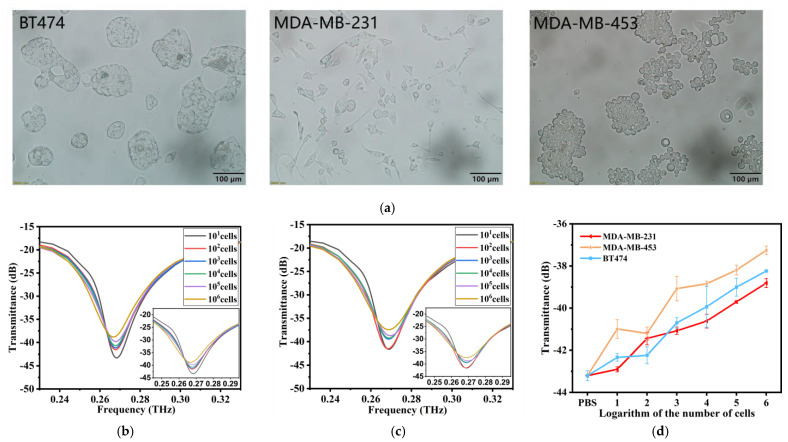
(**a**) Morphology of three cell lines after magnification under a microscope at by 200×. (**b**) THz transmission spectra of MDA-MB-231 cell solutions at cell numbers from 10 cells to 1 × 10^6^ cells. (**c**) THz transmission spectra of MDA-MB-453 cell solutions at cell numbers from 10 cells to 1 × 10^6^ cells. (**d**) The linear relationship between the cell numbers and THz transmittance of the three kinds of cells is compared. Error bars indicate the SD (n = 3).

As shown in Figure 4a, different breast cancer cells have different growth patterns and cell sizes, although they are all adherent and epithelioid cells. BT-474 cells are aggregated and grow in patches. MDA-MB-231 is dendritic. MDA-MB-453, which grows rapidly, has rounded cells that grow in clumps like grapes. The transmission spectra of MDA-MB-453 and MDA-MB-231 were obtained by adding them into the THz AR-HCF. We found that, similar to BT-474, with increasing cell number, the transmittance of the characteristic peak at 0.26 THz gradually increased, and there was a linear relationship, as shown in Figure 4b,c. Then, we compared the line plots of the transmission and the logarithm of cell number of the three kinds of cells at 0.26 THz and found that the transmissivity of different breast cancer cells at the same cell number was different, and the higher the cell number, the more obvious the difference was (Figure 4d). Therefore, the biosensors are capable of distinguishing different types of tumor cells through transmission spectra. The difference is mainly due to cell size and metabolism.

### 3.5. Cell Activity Analysis

**Figure 5:**
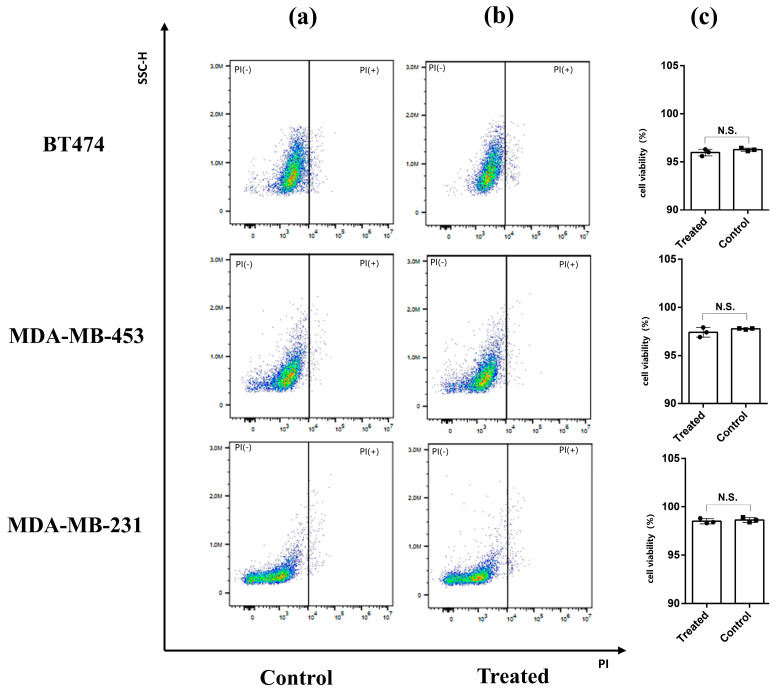
PI staining combined with flow cytometry was used to detect cell viability. (**a**) Cells not exposed to THz waves (control group). (**b**) Cells that were measured in the THz AR-HCF-treated group. (**c**) Cell viabilities of the control group and the treated group. Error bars indicate the SD (n = 3). N.S., not significant (*p* > 0.05).

After measurements were taken, all cell samples were collected and stained by PI. PI is a common red fluorescent nuclear and chromosome staining agent. Propyl iodide is also commonly used to detect dead cells in a population because it cannot penetrate living cells. When the sample was passed through the flow cytometer, the living cells did not produce a fluorescence signal, while the dead cells emitted red fluorescence under excitation by a 493 nm laser, which was used to distinguish the dead cells from the living cells. Figure 5a shows the negative controls (i.e., the results of the cell samples not exposed to THz waves). The cell viabilities of BT-474, MDA-MB-453, and MDA-MB-231 were 96.27 ± 0.16%, 97.76 ± 0.05%, and 98.63 ± 0.25%, respectively. Figure 5b shows the results of the BT-474, MDA-MB-453, and MDA-MB-231 cells that were irradiated by THz waves. The cell viabilities were 95.96 ± 0.35%, 97.40 ± 0.50%, and 98.5 ± 0.26%, respectively. Then, we analyzed the data via a one-way analysis of variance (Figure 5c). There was no statistical significance in the cell viabilities of the treated group and the control group of the three kinds of cells, indicating that the THz waves did not affect cell activity during the measurement. The sensor can enable the nondestructive detection of tumor cells. We speculated that the differences between the three cell groups might be caused by cell heterogeneity or the order of measurement because PI is slightly cytotoxic and can damage cell activity over time.

In addition to tumor cell detection, we believe that THz AR-HCF has potential for circulating tumor cell (CTC) detection. We compared the performance of our biosensor with state-of-the-art biosensors with respect to the detection of CTCs. Although the limits of detection of the THz AR-HCF biosensor are not as high as that of Raman spectroscopy, electrochemical sensors, and quartz crystal microbalances, the THz AR-HCF biosensor has a much higher linear detection range (Table 1). Other biosensors have a higher detection sensitivity than ours, but they require longer detection times, especially Raman spectroscopy. THz AR-HCF is capable of obtaining results in less than 10s, and unlike electrochemical and fluorescent biosensors, labeling is not required during use. Compared to other biosensors, THz AR-HCF has lower detection costs and shorter detection times. To provide a good foundation for the accurate identification of CTCs, an enrichment step is needed to increase the concentration of CTCs by several log units. Therefore, we believe that combining THz AR-HCF with CTC isolation and enrichment techniques such as microfluidic devices [34], Ficoll-Paque [35], and CTC identification using optical fiber after isolation and enrichment could enable the detection of CTCs in clinical samples.

## 4. Conclusions

In summary, we have developed a 3D-printed THz AR-HCF biosensor for the diagnosis and identification of tumor cells. Three-dimensional printing has the advantages of simple operation, low manufacturing costs, and fast production speeds. We tested different cell numbers of heterogeneous breast cancer cells and proved that this biosensor has good sensitivity and that it can distinguish different breast cancer cells based on THz transmission spectra. At the same time, the biosensor is capable of rapid and nondestructive detection, so it is compatible with sequencing, immunofluorescence, and even electrochemical biosensors and Raman spectroscopy to enable the multimodal detection of CTCs. Nonetheless, the proposed biosensor can only identify samples containing only one type of cell, not complex samples, which currently makes clinical sample analysis difficult. CTC detection in clinical samples not only requires extremely high sensitivity to detect several cells or even one cell in the sample, but also the exclusion of signal interference from other components, such as red cells and white cells, is needed in complex clinical samples.

## Figures and Tables

**Table 1:** Information on the current technologies for the detection of CTCs and the proposed biosensor.

Classification	Target Cell Line	Limits of Detection (Cells/mL)	Linear Ranges (Cells/mL)	Ref.
Fluorescence	MCF-7	100	100–1 × 10^5^	[36]
Surface-enhanced Raman scattering	HeLa	1	3–100	[37]
ICP-MS	MCF-7	87	250–10,000	[38]
Electrochemical	MCF-7	1	3–3000	[39]
Quartz crystal microbalances	MDA-MB-231	12	50–300	[40]
THz AR-HCF	BT 474, MDA-MB-453, MDA-MB-231	100	100–1 × 10^7^	-

## Data Availability

Not applicable.
