# Correction: He et al. A Novel Optical Fiber Terahertz Biosensor Based on Anti-Resonance for the Rapid and Nondestructive Detection of Tumor Cells. *Biosensors* 2023, *13*, 947

**DOI:** 10.3390/bios15110721

**Published:** 2025-10-31

**Authors:** Zhe He, Yueping Luo, Guorong Huang, Marc Lamy de la Chapelle, Huiyan Tian, Fengxin Xie, Weidong Jin, Jia Shi, Xiang Yang, Weiling Fu

**Affiliations:** 1 Department of Laboratory Medicine, Southwest Hospital, Army Medical University (Third Military Medical University), Chongqing 400038, China; hezhe957467915@163.com (Z.H.); yollowrong@sina.com (G.H.); marc.lamydelachapelle@univ-lemans.fr (M.L.d.l.C.); thylcq@163.com (H.T.); xiefengxin0310@163.com (F.X.); jinwd14@lzu.edu.cn (W.J.); 2 Tianjin Key Laboratory of Optoelectronic Detection Technology and System, School of Electronic and Information Engineering, Tiangong University, Tianjin 300387, China; 2131070920@tiangong.edu.cn; 3 Institut des Molécules et Matériaux du Mans (IMMM-UMR CNRS 6283), Université du Mans, Avenue Olivier Messiaen, 72085 Le Mans, France

## Text Correction

In the original publication [1], a correction has been made to Section 3.3, Paragraph 1. The revised version is as follows:

Figure 3d shows a good linear relationship between the logarithm of cell number and THz wave transmittance in the range of cell numbers from 10 to 10^6^. The linear fitting equation is T (Transmittance) = 0.8365Log(C) − 43.26, and the correlation coefficient is 0.9945.

## Error in Figure

In the original publication [1], there was a mistake in Figures 3d and 4d as published. We mistakenly wrote the cell-free sample as “lg0” and also wrongly included the cell-free sample in the fitting curve. The corrected Figure 3 and Figure 4 appear below.

## Figures and Tables

**Figure 3:**
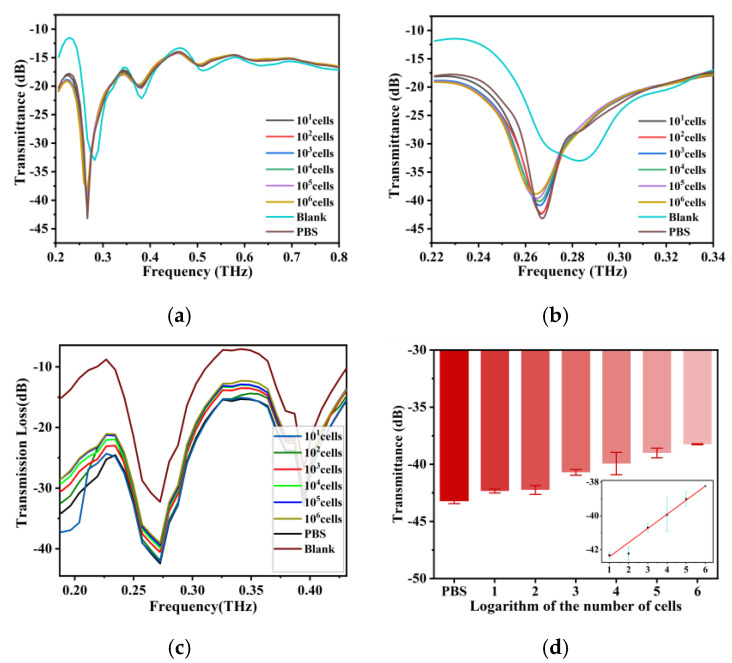
(**a**) THz transmission spectra of BT-474 cell solutions at cell numbers from 10 cells to 1 × 10^6^ cells and the THz AR-HCF without adding the sample between 0.2 THz and 0.8 THz. (**b**) Amplification of the signal from 0.22 THz to 0.34 THz of (**a**). (**c**) Simulation results of THz transmission spectra of fiber with cell numbers from 0 to 10^6^ BT-474 cells and without adding samples. (**d**) THz wave transmittance at 0.26 THz for BT-474 at different cell numbers. The inset shows the linear fit of the logarithm of cell number and THz wave transmittance. Error bars indicate the SD (n = 3).

**Figure 4:**
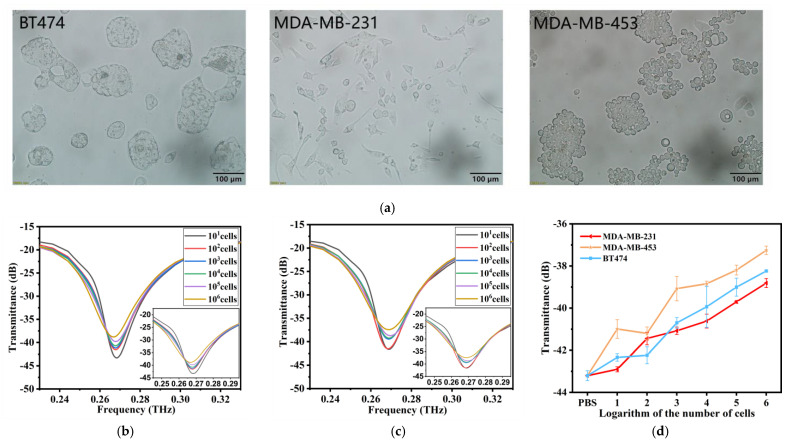
(**a**) Morphology of three cell lines after magnification under a microscope at by 200×. (**b**) THz transmission spectra of MDA-MB-231 cell solutions at cell numbers from 10 cells to 1 × 10^6^ cells. (**c**) THz transmission spectra of MDA-MB-453 cell solutions at cell numbers from 10 cells to 1 × 10^6^ cells. (**d**) The linear relationship between the cell numbers and THz transmittance of the three kinds of cells is compared. Error bars indicate the SD (n = 3).
